# Universal principles underlying segmental structures in parrot song and human speech

**DOI:** 10.1038/s41598-020-80340-y

**Published:** 2021-01-12

**Authors:** Dan C. Mann, W. Tecumseh Fitch, Hsiao-Wei Tu, Marisa Hoeschele

**Affiliations:** 1 Linguistics Program, The Graduate Center of the City University of New York, New York City, USA; 2 Department of Cognitive Biology, University of Vienna, Vienna, Austria; 3 Department of Psychology, University of Maryland, College Park, USA; 4 Acoustics Research Institute, Austrian Academy of Sciences, Vienna, Austria

**Keywords:** Animal behaviour, Human behaviour, Social behaviour, Language

## Abstract

Despite the diversity of human languages, certain linguistic patterns are remarkably consistent across human populations. While syntactic universals receive more attention, there is stronger evidence for universal patterns in the inventory and organization of segments: units that are separated by rapid acoustic transitions which are used to build syllables, words, and phrases. Crucially, if an alien researcher investigated spoken human language how we analyze non-human communication systems, many of the phonological regularities would be overlooked, as the majority of analyses in non-humans treat breath groups, or “syllables” (units divided by silent inhalations), as the smallest unit. Here, we introduce a novel segment-based analysis that reveals patterns in the acoustic output of budgerigars, a vocal learning parrot species, that match universal phonological patterns well-documented in humans. We show that song in four independent budgerigar populations is comprised of consonant- and vowel-like segments. Furthermore, the organization of segments within syllables is not random. As in spoken human language, segments at the start of a vocalization are more likely to be consonant-like and segments at the end are more likely to be longer, quieter, and lower in fundamental frequency. These results provide a new foundation for empirical investigation of language-like abilities in other species.

## Introduction

**Figure 1:**
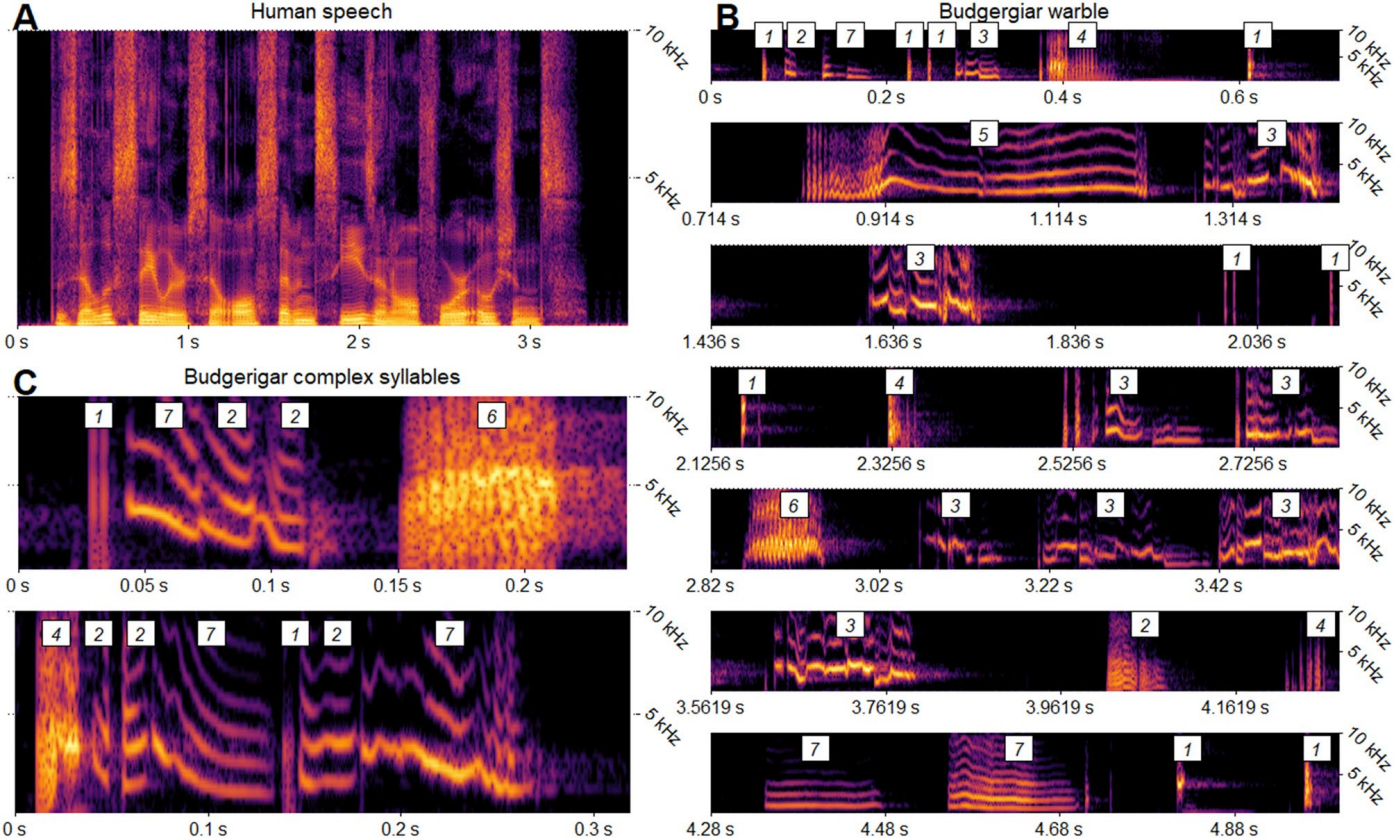
Complex vocalizations of the human and the budgerigar. In human language, silence is not a reliable cue for word, syllable, or segment boundaries. For instance, the complex and novel phrase “the zealous sailors sail all seven seas and all four oceans” can be spoken without any intervening silence, as shown in the spectrogram (**A**). Budgerigar song seems to share this property. Budgerigar song is comprised of complex (**B**: syllables 3 and 5) and simple (**B**: syllables 1, 2, 4, 6, 7) syllable types. The complex syllables (**C**), however, seem to be composed of segments which are similar to the simple syllables. We created the image using R and the packages *cowplot, ggplot2, ggpubr, seewave*, and *viridis*^19–23^.

One of the fundamental challenges in understanding animal vocal communication is delineating the units of production. These units are the basis for any study of vocal behavior as they underpin the critical data relevant to understanding within- and across-species variation, the mechanisms of vocal production, and functionality. In non-human vocal communication systems, units are typically defined relative to silences within the signal, with the most basic unit being uninterrupted sound^1^. While delineating by silence is useful, this approach may overlook important information, particularly when comparing a system to human language. In humans, a speaker can utter a long, complex phrase with no intervening silence, as in the phrase “the zealous sailors sail all seven seas and all four oceans”, seen in Fig. 1a. During speech, humans rapidly and actively modify tissue in both the vocal tract (e.g., tongue) and larynx (e.g., cricothyroid cartilages). Rapid shifts in one or more acoustic parameters result, and these shifts often mark perceptually discrete boundaries between units known as segments.

While the domain of syntax often receives the most attention when comparing human and non-human communication systems, research into segments and their organization (phonetics, phonology, phonotactics) is arguably the area of linguistics where our knowledge of cross-population uniformity and variability, and their underlying mechanisms, is most advanced. For instance, extensive cross-linguistic typological research has revealed that, in spite of the great diversity of languages, all spoken languages have two broad classes of segments: plosives, transient bursts of energy (e.g., *p*, *d*, *k*), and vowels, periodic signals with clear harmonic structure that are typically made with little to no vocal tract obstruction (e.g., *i*, *u*, *a*)^2–5^. In spite of cross-population similarities in low-level units, humans can achieve high degrees of diversity in sound systems because of the ability to combine and rearrange a limited set of units to create a functionally infinite set of larger units (like syllables, words, and phrases). The ability to generate novel units from low level elements had been cited as a trait that separates humans from other animals: animal repertoires were finite and static while human language needed a generative system to create a vast inventory of symbolic labels^6,7^.

However, there is ample evidence that species of parrots, songbirds, and whales can create novel songs by combining and rearranging breath groups or “syllables”^8,9^. The precise evolutionary function of these generative systems and whether the ability holds for multiple levels, as it does in humans, however, is unclear. Because humans are the only species for which there is evidence of generativity below the level of the breath group and are also the only species where there is evidence of using vocal signals to convey complex symbolic meaning, a connection between the two phenomena seems logical^10^. But because we lack the data for segments in non-humans, this connection is presumptive. Non-generative segment systems seem to exist in non-vocal learners like banded mongooses^11^. Furthermore, some species of songbirds, like the grey catbird, have vast syllable repertoires, suggestive of a generative system for syllable creation^12^. In addition, the physiological gestures that zebra finches use to produce song do not correspond to the silent intervals surrounding syllables, suggesting that the finches need to combine multiple motor movements to produce one syllable^13^. These motor movements could mark segment boundaries or they could be similar to the articulatory gestures that build segments in spoken human language where there is a complex, learned relationship between physical gestures and the perceptual segment categories (phonological alternations, historical and synchronic, often affect segments classes that share motor gestures). In other words, the zebra finch gesture data^13^ and the data we will show here are akin to the parallel tracks of phonetics and phonology, respectively. Understanding segments in non-humans is essential for our understanding the relationship between complex acoustic communication and meaning as well as the evolution of vocal learning and language.

One species that has great potential for segmental analysis is the budgerigar, a small parrot native to the arid regions of Australia. In their socially learned song (often called “warble”), “exact repeated renditions” of complex syllables are incredibly rare^14^. Budgerigars are a fascinating model species because they can mimic human speech and they have perceptual traits once considered unique to humans. They can distinguish human words based on stress patterns (*puVO* vs. *PUvo*) and they can categorize human segments by using linguistic cues rather than individual speaker cues (e.g., *a* is treated the same when spoken by people of difference age, sex, size, etc.)^15,16^. They also have song that is learned, complex, and highly variable within and across different populations, much like human speech. However, more attention has been given to their human speech perception abilities than their complex song.

Only a few studies have analyzed budgerigar song, likely due to the lack of stereotypy. The few studies which have analyzed budgerigar song have found broad syllable categories, some of which are simple and highly stereotyped—such as “alarm call-like” and “click” syllables—while others are complex and non-stereotyped—“contact call-like” and “compound” syllables ^14,17^. From a visual inspection, complex song syllables seem to be composed of subunits which are potentially more invariant and stereotyped (see Fig. 1). These subunits have not been analyzed in budgerigar song, likely due to the difficulty in dividing the acoustic stream by something other than silence. Manual segmentation can be subject to human biases and can be functionally impossible with a large amount of data. To help overcome these issues, we designed an algorithm in Praat^18^ which automatically divides song syllables into segments.

If segments are a unit of production in budgerigar song which can be used to build larger units, we expect to find certain patterns. We predict that segments should cluster better and show less variation across individuals and populations when compared to syllables. Based on research in spoken human languages, we also expect to find biases in how segments are organized. Specifically, we expect segments at syllable edges to be lower in fundamental frequency, longer, less periodic, and quieter than the same segments produced in the middle of syllables.

## Results

To have a starting point for the algorithm, we used the vast array of knowledge of the species where we know most about segments: humans. The human data also permits the ability to directly compare budgerigar vocalizations to human speech. We created the algorithm using a broad sample of human speech that spanned the diversity of both language and sex differences in vocalization. This was done to create an effective species-level segmentation algorithm in humans. Because silence is not the only acoustic cue to segment boundaries in human speech, the algorithm uses rapid transitions in fundamental frequency, amplitude, and/or spectral dispersion (Wiener entropy) to mark segment boundaries. Only after validating the algorithm on humans did we apply the algorithm to budgerigars (see “Methods”).

**Figure 2:**
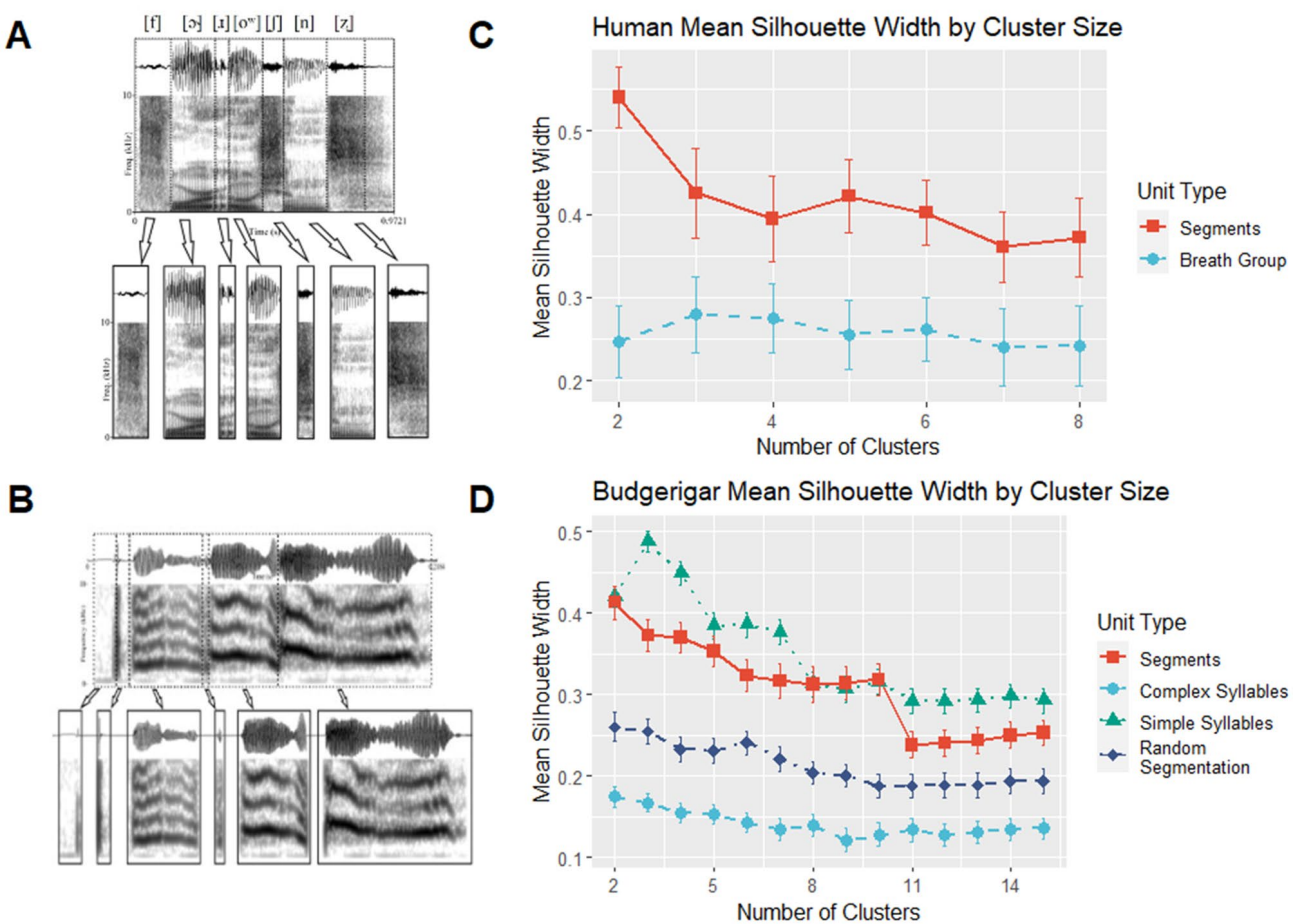
Segmentation and segment validation. (**A**), an example of an output of our segmentation algorithm on human speech, in this case, an English vocal breath group, “four oceans”. (**B**), an example the segmentation algorithm applied to budgerigar song. We scaled the algorithm to the different species by making the algorithm dependent on a minimum fundamental frequency setting. For each cluster size of human speech (**C**), silhouette widths were higher for segments than for syllables ($\bar{x}$ = 0.42–0.25). For the budgerigar units, we included simple syllables and random snippets of syllables that were equally long as the segments as controls (**D**). Silhouette width scores for segments were statistically different from complex syllables ($\bar{x}$ = 0.32–0.13, *V* = *105, p* < *0.001*) and the random snippets ($\bar{x}$ = 0.32–0.22, *V* = *105, p* < *0.001*). We created the image using Praat, R, and the R packages *ggplot2* and *magick*^18,19,25,26^.

We applied the algorithm to song from 14 budgerigars from 4 independent populations. To validate whether segments are a unit of production in budgerigar song, we clustered segments and syllables and assessed cluster discreteness by calculating silhouette widths—a measure for how well data points fit within their cluster compared to how well they would fit in the next nearest cluster (see “Methods”)—for various cluster sizes. We found that segments produced much more discrete clusters when compared to complex syllables (see Fig. 2). As a second validation method, we tested how reliable the acoustic cues present in segments and complex syllables were at predicting individual or group identity. In a particulate system, basic units are more discrete and invariant than the units that they build. To illustrate, a human phrase, like the one in Fig. 1, may have been produced only a handful of times throughout history and is immediately recognizable as English. However, the segments within the phrase are far more stereotyped and widespread; coronal nasals, like the sound [n] in *oceans* (Fig. 2: [oʷʃnz̥]), are present in over 3/4ths of all spoken human languages and broad segment classes, like vowels, nasals, or plosives are even more widespread^5,24^. If syllables are the most basic unit, information extracted from segments and syllables would be redundant. We found that budgerigar syllables are more reliable than segments at predicting group (66–48%) and individual identity (38–25%). Because both of these results suggest that segments are more discrete and invariant than syllables, our segmentation algorithm was able to accurately divide budgerigar syllables into segments. This suggests that segments in budgerigars may be similar to those of humans in that they are more likely to occur across populations than larger units.

**Figure 3:**
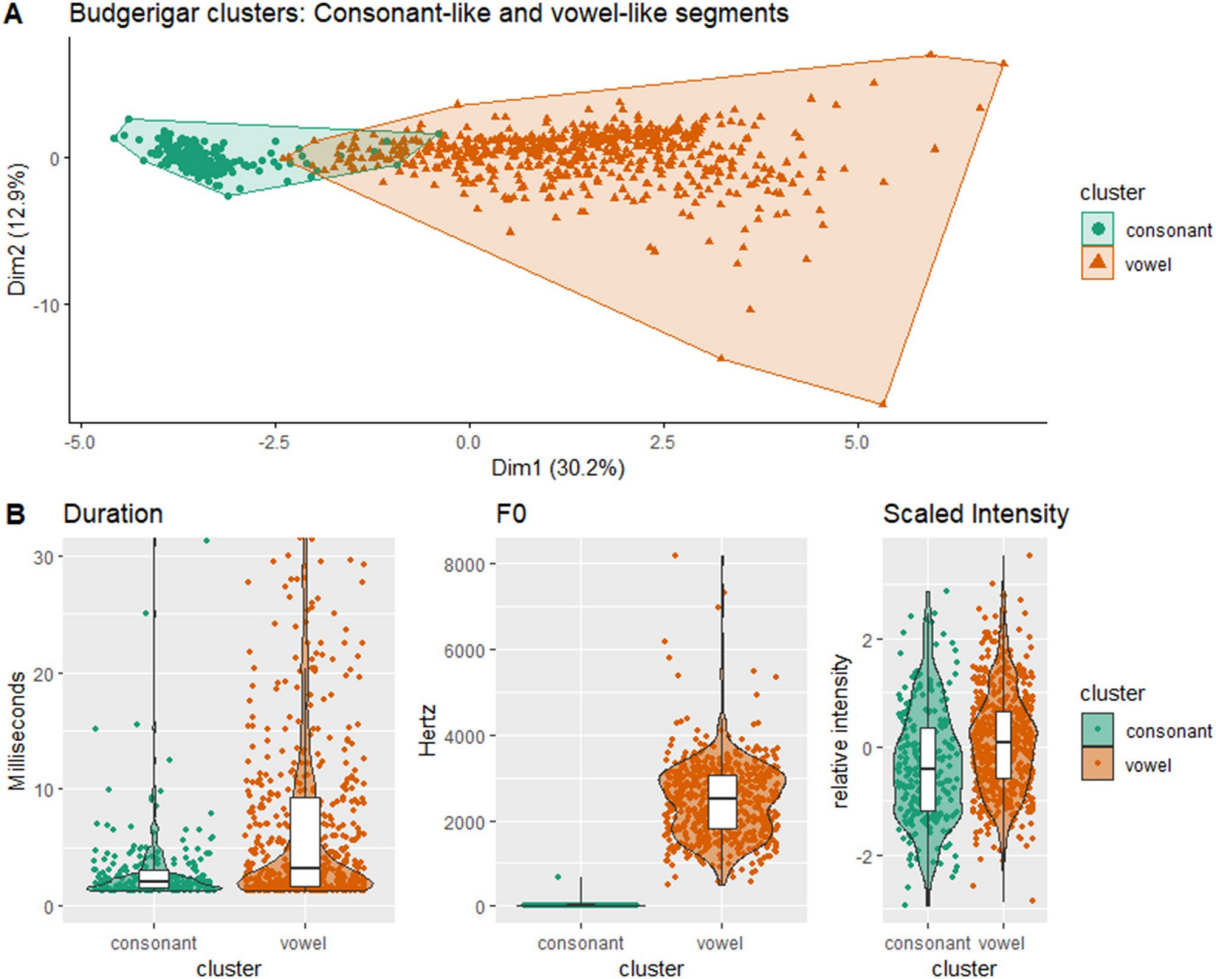
Budgerigar vowels and consonants. (**A**) A hierarchical clustering of budgerigar segments reveals two clear clusters of segments. (**B**) Using the clustering, we found that one cluster is comprised of periodic, vowel-like units while the other is made up of aperiodic, consonant-like units. The former is, on average, longer, louder, and more periodic than the latter. We created the image using R and the packages *factoextra* and *ggplot2*^19,25,27^.

As shown in previous research, budgerigars can rearrange and combine syllables to create a vast repertoire of song types. Because segments can also be combined and rearranged within syllables, our data show that a non-human can generate novel acoustic signals at multiple levels. Furthermore, the broad segment classes that came out of our hierarchical clustering suggest parallels between budgerigar segments and human language segments. Using the functions *eclust()* and *fviz_silhouette()* from the R package *factoextra*^27^, we found that a cluster size of 2 produced the most distinct clustering (as measured by silhouette widths; Fig. 2). These two clusters differ in length, intensity, and the presence or absence of a clear fundamental frequency (Fig. 3). This division in budgerigar segments parallels the consonant–vowel dichotomy in spoken human languages. Budgerigars segments are divided into segments with a clear fundamental frequency and harmonic structure, “vowels”, and segments where the acoustic signal is noisy or chaotic and there is no discernable fundamental frequency, “consonants”.

In practically every spoken language, speakers contrast periodic signals with clear harmonic structure (modally voiced vowels) and bursts or noisy segments lacking in a clear F0 (voiceless plosives and fricatives, or “obstruents”)^2,24^. Technically, human vowels can be unvoiced (e.g., whispered vowels lack an F0) and consonant sub-classes vary considerable (including some consonant classes, like glides, that are very vowel-like in their acoustic qualities); but, voiced vowels and voiceless plosives/fricatives are the most common segment classes and very few, if any, spoken languages exist that do not contrast these sound classes.

In spite of human flexibility in segment recombination, segment organization across languages is not symmetrical. In fact, similar organizational patterns have been found at phrase boundaries across a wide range of historically unrelated languages^28^. For instance, languages which permit syllable-initial sound sequences like [ka] greatly outnumber those which permit a reversal of those sounds, e.g., [ak] (related to the consonant–vowel (CV) preference and the Margin Hierarchy)^29–33^. If our segmentation algorithm was successful and budgerigars have organizational biases, we should see differences at syllable edges.

We divided the budgerigar vocal segments into three categories based on their relative position in the syllable: initial, medial, and final. We then assessed if all fourteen individuals across all four populations shared similar edge preferences in four acoustic parameters: fundamental frequency, periodicity, duration, and intensity. In humans, segments at the end of an utterance are longer, lower in fundamental frequency, lower in amplitude, and often have reduced periodicity. In initial positions, periodic signals are less common. Medial segments are often associated with loud, periodic signals. If segments in budgerigars are similar to human vocal segments, we expect to find similar patterns. That is, we expect syllable-medial segments to have higher proportions of periodic segments and syllable-initial segments should be the most aperiodic; we also expect that final segments will have lower amplitude, lower fundamental frequency, and longer duration compared to medial segments.

**Figure 4:**
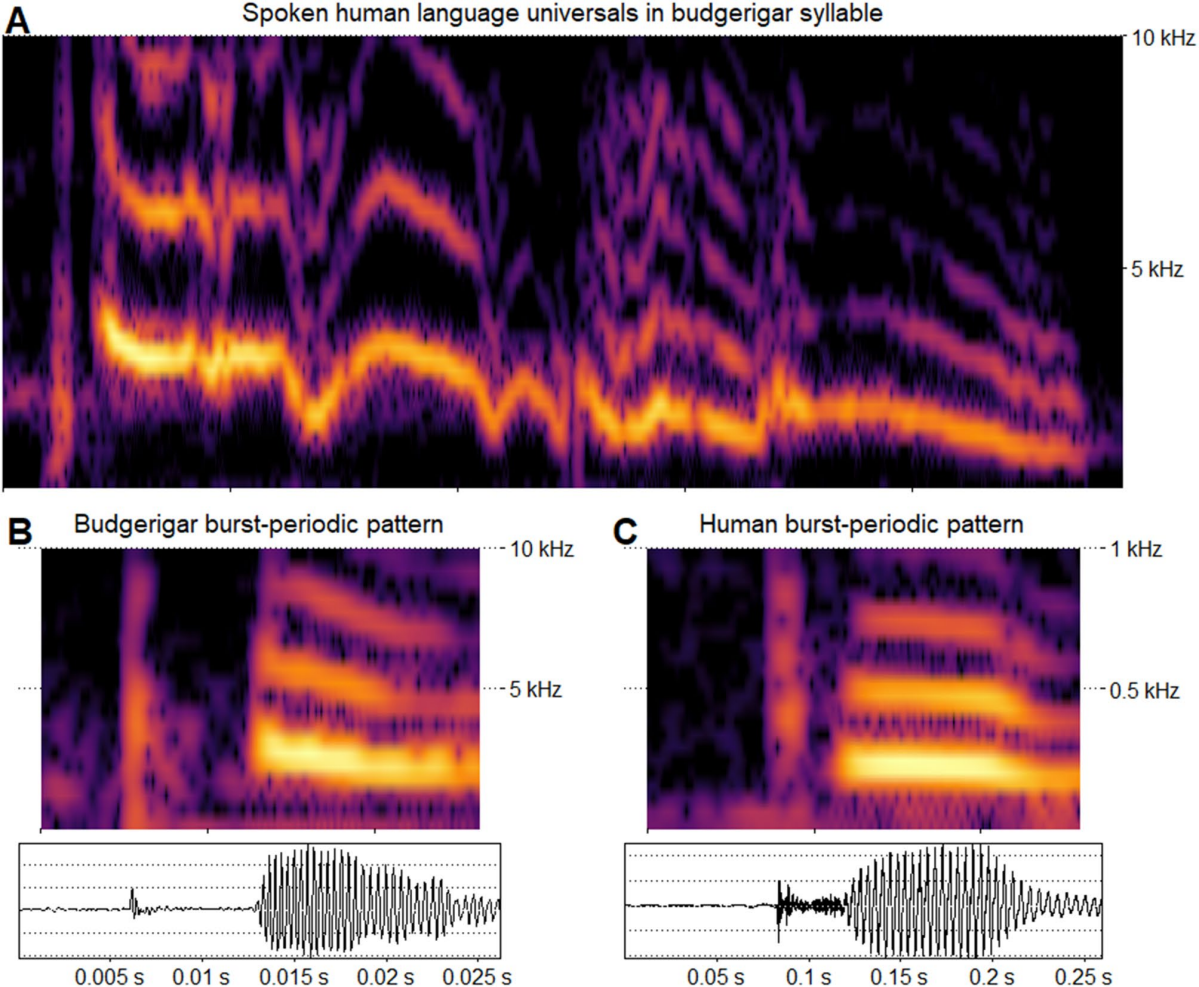
Edge effects in budgerigar song. (**A**) Canonical syllable with aperiodic burst onset, long and low final segment, and a rise-and-fall intensity contour. All groups shared similar positional biases. For all individuals, mean F0 measurements were lower for segments in syllable-final position when compared to medial segments (mean of individual means: *n* = 14, Medial: $\bar{x}$ = 2522 Hz, *s* =  ± 181 Hz ~ Final: 2069 ± 165); final segments were, on average, longer in duration than medial segments (*n* = 14, Medial: $\bar{x}$ = 6.5 ms, *s* = 0.84 ms ~ Final: 12, ± 4.84); intensity was lowest in initial position (Initial: $\bar{x}$ = 46.7 dB *s* = 3.71 dB ~ Medial: 56.4 ± 4.03 ~ Final: 52.8 ± 4.46); and periodicity was lowest in initial position with slightly more than a third of segments having periodic vibration (*n* = 14; $\bar{x}$ = 34.3%, *s* = 10.5% ~ Medial: 74%, ± 4.9 ~ Final: 54.9%, ± 10.5%). For all four acoustic measurements, a mixed-effect model with position as a fixed effect performed better than a model without position (F0: *X*^*2*^ = *2353.4, df* = *2, p* < *0.001*; duration: *X*^*2*^ = *3209.6, df* = *2, p* < *0.001*; intensity: *X*^*2*^ = *22,186, df* = *2, p* < *0.001*; periodicity: *X*^*2*^ = *10,158, df* = *2, p* < *0.001*). (**B**) The prevalence of aperiodicity in syllable-initial positions and periodicity in syllable-medial positions suggests that budgerigars share the human segment organizational preference (**C**) for aperiodic onsets followed by periodic signals. We created the figure using R and the packages *cowplot*, *ggpubr*, *seewave* and *viridis*^20–23^.

Not only did we find similarities across all four populations of budgerigars (Fig. 4) these patterns were consistent with segment organization biases in human language. In humans, segments at the end of a breath group tend to be longer in duration and lower in fundamental frequency and intensity^34,35^, while initial segments are less periodic than medial and final segments^28^.

While positional dependent differences at the level of the segment have not been described in any other species, there is evidence that some of these patterns are somewhat common at higher levels of analysis. Songbird syllables are on average longer at the end of a song bout than song medial syllables^36^. Also, F0 tends to decrease at the end of a song. In two-note black-capped chickadees calls, the second “note” (equivalent to a breath group or syllable) is lower in intensity, especially for subordinate males^37^.

Because these patterns are found across several species, the underlying mechanisms are likely to be based in general sound production or perception phenomena. For instance, the mechanisms underlying lower F0 and amplitude in final position seem to be directly related^38^. Air volume decreases throughout the production of vocal units, particularly longer and more complex units like utterances, songs, or phrases. A decrease in air volume will lead to a decrease in amplitude, all else being equal^39^. This decrease in air volume will also affect subglottal pressure, which is one of the determining factors of the rate of vocal fold vibration^40,41^. Final lengthening may be the result of gradually slowing articulators in preparation for the end of a vocalization^42,43^. Humans and songbirds rapidly adjust their articulators during vocal production and abrupt termination of these movements may be more difficult than a gradual relaxation of articulators^36,43^. Because budgerigar segments are produced within a single breath, unlike the songbird units, the data presented here are even stronger evidence in support of widespread bio-mechanic mechanisms.

We also found a pattern that has not been described at any level in non-humans until now: the CV preference, the preference for consonants to precede vowels in syllable-initial positions. In humans, the underlying mechanism for this pattern is not largely agreed upon. Language and human specific-hypotheses for the CV preference have been proposed (e.g., Universal Grammar^33^). While we cannot rule out a coincidental relationship without further research, the similarity between budgerigars and humans suggests that species-specific explanations are not warranted and that we should consider (one or more) widespread cognitive, functional, perceptual, or bio-mechanical mechanisms. For instance, one possibility is the maximization of contrastive units^4,44^. When taking the capabilities of the human vocal tract and ear into consideration, aperiodic bursts (the only consonant class that may be universal in spoken languages^2^) and periodic signals (with harmonic stacks) are the sound classes that are the most distinct from each other. By using sounds from these two classes, humans can maximize the amount of auditory space and minimize confusion for the listener. Furthermore, a burst followed by a periodic signal seems to be better than the reverse pattern at preserving the acoustic cues of both segments^45,46^.

For budgerigars, bursts and periodic signals likely occupy opposite ends of the budgerigar articulatory-acoustic space, as well. Presumably, whatever acoustic cues are relevant to the budgerigar system should be preserved with a burst-periodic pattern as well. Though it is less clear why budgerigars would need to maximize perceptual distinctiveness, particularly since there is no evidence for a large symbolic inventory as in human language. The function of song is not completely understood, though it is clearly relevant in courtship^47–49^. If females prefer more diverse signals, the males may use a burst-periodic pattern to maximize the chance that their diverse repertoire is noticed. However, the evidence for repertoire size being the result of sexual selection is less robust than once believed^50^. Specifically in budgerigars, Tobin, Medina-García, Kohn, and Wright^51^ found that female-directed song is actually more stereotyped than male-directed song with respect to phrase “syntax”. The social environment that budgerigars typically sing in also does not seem conducive to the preservation of the aperiodic bursts. Multiple individuals sing at the same time in close proximity to each other; in the wild thousands of budgerigars could be in one area at the same time^52^. Aperiodic sounds, when compared to periodic sounds, are less robust in noisy environments; in human speech, cues to bursts can even be masked by surrounding speech sounds^46^. That being said, the onset of the breath group is the most optimal position to minimize masking from ambient noise and their own periodic signals, which is consistent with our data.

Another (non-mutually exclusive) possibility is that budgerigars and humans share sound production mechanisms which lead to phrase initial bursts. In numerous languages, reduced periodicity and/or periodic bursts occur, even when speakers and listeners don’t perceive the sound (e.g., *glottal stop insertion*^28,53–57^). This is particularly true at the beginning of a phrase and with emphasis^28,54^. For instance, English speakers will often produce a glottal stop before the vowel in a word like *apple* or *issue*, though most listeners don’t recognize that it is present^54^. In humans, unless other measures are taken, vocalizations will naturally begin with a brief interval of aperiodicity^56,58^. The budgerigar and human patterns could be the result of the difficulty in initiating voicing directly from non-phonation. With a closed vocal tract the pressure variation above and below the vibrating tissues is not sufficiently different for phonation; once the closure is released and the appropriate pressure differential can be reached then voicing can begin^58^.

It is premature to assume that budgerigars and humans are the only species who would share the CV preference. While this pattern hasn’t been described as widespread or systematic for any other species, published descriptions or spectrograms suggest that at least some populations of species may have somewhat similar tendencies^11,59^. Without a segmental approach, like the one presented here, this pattern is likely to go unnoticed. Furthermore, comparative research should help us understand whether the CV preference in humans and budgerigars (and potentially other species) emerges from common functional, perceptual, or bio-mechanical pressures or is only superficially related and the result of wholly distinct processes.

Our data are consistent with research in zebra finches which has found evidence of “gestures” within syllables^13^. Research on the neurobiology of birdsong had found no clear relationship between the firing of neurons in the HVC—an area of the songbird brain that shows large amounts of activity during singing—and syllable boundaries, leading some to suggest that HVC neurons fired at regular intervals^60^. Amador and colleagues, however, found that neural firing matched with articulatory gestures, defined as shifts in air sac pressure or syringeal labia tension^13^. Our results show that we can recover some of these gestures by using less invasive spectrographic data. The relationship between gestures and segments, however, is unclear. In humans, segments are built from multiple articulatory gestures in both the sound source and filter. The vocal apparatuses in songbirds and parrots are distinct from each other and from the human vocal apparatus; still, there is ample evidence that species of songbirds and species of parrots modify their acoustic signal by actively controlling articulators in the sound source and vocal tract^61^. Taken together, our results support the idea that syllables are not necessarily the basic unit of vocalization in non-human species. Rather, we propose that there are structural similarities with spoken human language where articulatory gestures (shown in zebra finches) compose segments (shown here), which, in turn, combine to build syllables, words, and phrases (the usual level of analysis in animal vocalizations).

To date, there is no evidence that budgerigar song has the referential aspects of human language, as such, it is an enticing model for a “bare phonology” and may even be able inform on more difficult questions related to human language evolution^62^. Studdert-Kennedy^7^ and Jackendoff^63^ have argued that the ability to combine and rearrange discrete units may have been an important step in the evolution of language by allowing humans to apply a label to practically any object, event, idea, or proposition. They argue that holistic phrases quickly lose their perceptual distinctness as the inventory grows. However, if song does not have the referential aspects of human language, the pressure to create novel labels may not be a requirement for the development of particulate segment system.

Budgerigar song is sung mostly by males and, in part, during courtship^47^. The similarities in segmental systems of humans and budgerigars are also consistent with Darwin’s “musical protolanguage” hypothesis of human language evolution, which proposes that speech and vocal imitation first developed to support courtship displays similar to the song of many whales and temperate zone songbirds^62,64,65^.

In summary, despite major differences in physiology, habitat, phylogeny, and function, budgerigars and humans independently evolved segmental structure. The presence of similar acoustic units, and similar regularities over these units, in these two species provides a comparative opportunity to assess the pressures which give rise to complex acoustic communication. Furthermore, claims about what aspects of human language non-humans possess often rely on comparisons between very different types of analyses. Segmental analyses provide a novel and promising basis to further investigate whether human linguistic phenomena like natural classes, coarticulation effects, and phonological rules also exist in animal systems^66^.

## Methods

### Data collection: budgerigar song

We segmented and analyzed the vocalizations of a total of fourteen budgerigars, thirteen males and one female, from four independent populations (groups A, B, C, and D). Individuals from groups A and B were recorded in their aviaries (Group A: 2.5 × 2 × 2 m; Group B: 2 × 1 × 2 m) in the Department of Cognitive Biology at the University of Vienna. The aviaries are located in separate, non-adjacent rooms at the university. Group B shares a room with another aviary with which it has acoustic, but not visual or physical, contact (no individuals were recorded from this other group). The rooms of both A and B are lined with acoustic foam padding (Basotect 30 Plain) to reduce echo and outside noise. The colony from which group A was recorded has a total of 12 budgerigars, six of which are male. The colony of group B has six individuals with three males. We were able to record seven individuals from group A and one from group B.

Group C is comprised of two pet budgerigars who were recorded at a home in Arkansas, USA. They were recorded in a metal wire cage (70 × 60 × 50 cm) lined with the same acoustic foam as with groups A and B.

We habituated groups A, B, and C to the presence of a human with recording equipment in their social environment and then opportunistically recorded individuals so that we could record song that is as close to their naturalistic performance as possible. These groups were recorded with an H4N Zoom recorder and a Sennheiser directional shotgun microphone at a sampling rate of 44.1 kHz. We mounted a GoPro Hero 4 to the top of the shotgun microphone. We recorded video (30 frames/sec) during the recording sessions in order to precisely identify the vocalizing individual.

The final four individuals (Group D) were recorded at the Laboratory of Comparative Psychoacoustics at the University of Maryland. The recordings of three of the individuals in group D were from archival recordings presented in Tu^67^, Tu et al.^17^, and Tu and Dooling^68^. The final individual from Group D was recorded ten years later. All individuals were recorded under the same conditions; recording details can be found in Tu^67^, Tu et al*.*^17^, and Tu and Dooling^68^.

### Data collection: spoken human language

Because no data currently exist for budgerigar segments, we used human speech to guide research into budgerigar song. We used vocalizations from five historically unrelated languages: Chickasaw, Georgian, English, Vietnamese, and !Xóõ. We chose these languages based on a combination of language relatedness, access to audio files with phrases, good signal-to-noise ratio, and speaker sex (Female: Chickasaw, Georgian & English; Male: !Xóõ, English, & Vietnamese). All these factors serve to increase the diversity of acoustic signals, which is important because we were interested in studying segments in human speech at the species level, rather than at the language level. For example, males have, on average, a lower fundamental frequency than females, !Xóõ has one of the largest segment inventories across all languages, Vietnamese and Chickasaw use F0 to differentiate words, and Georgian uses complex sound sequences. These factors also help to prevent overfitting a segmentation model to a specific language, language family, sex, or individual. With the exception of one English speaker, the files were collected from the UCLA Phonetics Lab Archive^69^. In the archive, each sound file is accompanied by recording details and transcripts. Most of the sound files have speakers uttering short words, so we specifically looked through the transcripts for those that had longer utterances.

In linguistics, “syllable” refers to an organizational unit of segments rather than a vocalization separate by silence as is common in animal communication. To be explicit, for the segmentation of human vocalization, we used vocalizations separated by silence, the breath group.

We used three English speakers. The first two were taken from the Vietnamese and Georgian sound files. In these files, English speakers often prompted phrases or described what was occurring in the recordings. The English speaker in the Georgian file was a native English-speaking female. In the Vietnamese file, the speaker was male and a non-native speaker, likely of Vietnamese, though the recording notes did not make the native speaker’s linguistic background explicit. The final English speaker was the first author, a native speaker of American English. Those utterances were recorded in a semi-anechoic room at the University of Vienna using an H4N Zoom recorder at a sampling rate of 44.1 kHz. As a further validation, we used recordings from the Arabic Speech Corpus^70^, a corpus of spoken Arabic that includes broad phonetic transcriptions of the speech.

### Data collection: ethics

All procedures performed in animals were conducted in accordance with local animal protection and housing laws and were approved by the ethical board of the behavioral research group in the faculty of Life Sciences at the University of Vienna (groups A, B, and C) or the Animal Care and Use Committee of the University of Maryland, College Park (group D). For the recording of the human subject, we obtained informed consent for publication of any identifying recordings in an online open-access publication. The procedures were approved by the University of Vienna Ethics Committee. The other recordings were collected from two publicly available databases, UCLA Phonetics Lab Archive^69^ and the Arabic Speech Corpus^70^.

### Data preparation: syllable extraction from budgerigar song

From the recordings of the budgerigars we extracted bouts of song using a custom Praat script. The script used Praat’s *Annotate: To Textgrid (silences)* function to label sections of the recording as potential song bouts. We used − 45 dB for the amplitude threshold and one second duration for the threshold for silence, meaning that if amplitude was less than 45 dB down from the peak amplitude for longer than one second, the section was labeled as silence. The rest was labeled as a vocalization. Vocalizations that were longer than 2.5 s were labeled as song. We manually coded those sections by individual and quality. To code individual, we cross-checked with the recording notes and video files. For quality, we excluded bouts where two or more individuals were vocalizing simultaneously, and we could not determine which vocalizations belonged to which individual. In some cases, we were able to extract sections of one individual vocalizing from these longer, multi-vocalization bouts.

We ran each song through another custom Praat script that divided the songs into syllables. The script used a pass Hann band filter (*Minimum frequency: 1 kHz, Maximum frequency: 15 kHz, Smoothing: 100 Hz*) to exclude any noise outside of the typical budgerigar song range. It then created an intensity envelope by calculating the root-mean-square (RMS) of the sound pressure (window duration: 25 ms; time step: 5 ms). The algorithm identified syllables by checking for intervals where sound pressure RMS dipped below 1/6th of the song sound pressure RMS for longer than 10 ms.

Our algorithm labeled syllables based on the Tu et al*.*^17^ classification. We collapsed the compound and contact call-like syllables into a single “complex syllable” category. These complex syllables were extracted for segmentation.

### Segmentation algorithm: developing a method of automatic breath group/syllable segmentation

For automatic syllable segmentation, the algorithm took multiple measurements at regular intervals throughout the vocalization for amplitude, fundamental frequency, and Wiener entropy. We made the algorithm easily scalable between humans and budgerigars by making measurement windows and sampling intervals dependent on a species minimum fundamental frequency. Minimum human F0 was set at 50 Hz and budgerigar F0 was set at 400 Hz, roughly the bottom F0 range for each species. For each acoustic measurement, the algorithm calculated the percent change between the acoustic measurement and the subsequent measurement. A percent change greater than a predetermined value marked a segment boundary. (The specific magnitudes are discussed below.) A second pass searched for smaller magnitude changes that are correlated between intensity and either F0 or Wiener entropy. That is, a smaller scale change in amplitude may mark a segment boundary if within the same time window, a small change in F0 or Wiener entropy is also present. The size of the window is determined by dividing 0.5 s by the minimum species F0. We didn’t include correlations between F0 and Wiener entropy because a change in F0 is necessarily correlated with a change in Wiener entropy. Finally, to prevent the insertion of multiple boundaries associated with the same change, we added a buffer. The buffer is the same duration as the window size for correlations between acoustic changes, e.g., in a human vocalization, a boundary cannot be inserted within 10 ms of another boundary (0.5 s/50 Hz).

### Segmentation algorithm: human breath group test

We first tested the algorithm on human speech, a communication system where we can evaluate the output. While technology related to speech parsing has become advanced and highly accurate, these algorithms and models use pre-segmented or labeled training sets, data that is unavailable and unknowable (at this point) for budgerigars. That being said, because human speech and budgerigar vocal behavior makes use of similar acoustic principles (e.g., pulmonic expiration, myo-elastic dynamic theory, source-filter theory, etc.), a segmentation algorithm that can approximate true segment boundaries in humans is useful for detecting potential boundaries in the acoustic signal of budgerigars^61,71^.

However, even human speech presents difficulties. Boundaries can be unclear and speech can be highly variable. The physical realization of a perceptual unit is not completely identical each time it is produced, furthermore, the physical form may vary considerably depending on context. Spoken languages also vary in the acoustic features which mark meaningful boundaries. These features may still be present in a language even if they don’t use them to mark meaning. For instance, a speaker may switch from regular phonation to slightly irregular phonation during the production of a vowel. English speakers do this, though often do not recognize it. A speaker of Jalapa Mazatec, where phonation types distinguish meaning, would recognize the shift as a change in vowel type, however^72^. When analyzing human speech, researchers often must make choices about which acoustic features to ignore as many may be irrelevant to their research question or language of interest. These decisions are greatly aided by an understanding of what is perceptually relevant to the speakers.

Because we don’t yet have a basis for perceptual segments in budgerigars, we decided to segment the human signal without relying on potential phonological representation, therefore, we did a narrow phonetic transcription and segmentation using the conventions laid out in Keating, et al*.*^73^ for each human language phrase. To avoid overfitting a model to one language and to prioritize generalizability (relative to accuracy), we built the algorithm using transitions in only three acoustic features: fundamental frequency (F0), amplitude, and Wiener entropy. These three acoustic features were used because they can also be measured in budgerigar vocalizations. We then removed transitions that are based primarily on formant transitions (e.g., vowel-vowel, approximant-vowels). Formant transitions are useful for most human languages, but because of the short vocal tract and high fundamental frequency, formants are not likely to be a robust cue in budgerigar vocal behavior.

After optimizing the input settings, we set large transition values of 6 dB per frame for amplitude, 190 Hz per frame for F0, and 150 per frame for Wiener entropy. For smaller, correlated transitions the values were 2 dB per frame for amplitude, 7 Hz per frame for F0, and 90 per frame for Wiener entropy.

We assessed the accuracy of the segmentation by using the package *rPraat*^74^ to read Praat TextGrid files into R^19^. The TextGrid files contain time stamps of the “true” manual segmentation with the automatic segmentation. Each file was divided into frames of 25 ms which we used to check the presence or absence of both manual and automatic segment boundaries. We used the R package *InformationValue*^75^ to calculate the accuracy, sensitivity, and specificity of the automatic segmentation. For the initial data set, the test on fully segmented speech resulted in an accuracy rate of 79% (sensitivity = 50%, specificity = 89%). For non-format transition (e.g., obstruent-vowel sequences), the algorithm achieved an accuracy rate of 86% (sensitivity = 70%, specificity = 90%). To ensure our results were not only valid for our initial data set, we tested speech samples on a novel data set. We gathered a random sample of 5 wav files, with phonetic transcriptions, from the Arabic Speech Corpus^70^. As with our own transcription, we removed boundaries based on formant transitions. With the novel dataset the algorithm scored 73% (sensitivity = 44%, specificity = 83%) for the full segmentation and 81% (sensitivity = 64%, specificity = 84%) for non-formant transitions. We don’t expect to be highly accurate with human speech (or budgerigars, necessarily), but our scores are somewhat comparable to other methods^76,77^ We used the same input values to the algorithm for budgerigar syllables.

### Segmentation validation: clustering of human vocal units

We performed a cluster analysis on human language data so that we would have a clearer idea of what to expect from the results of the budgerigar song segmentation. We used the same recordings described earlier.

We took fifteen random vocalizations from each of the seven speakers. As in the previous case, we manually segmented the vocalizations based on the guidelines in Keating et al*.*^73^, though in this case we did not remove boundaries defined by formant changes. We then broadly labeled each segment for manner (vowel, glide, approximate, nasal, fricative, affricate, stop, and click), place of articulation (labial, coronal, palatal, velar, glottal, high/mid/low, front/central/ back), and whether the segment was voiced or voiceless, nasalized, glottalized, a rhotic, a lateral, a tap, or a trill.

We ran the vocal breath groups and segments through a Praat script which extracted acoustic parameters. We chose only a few parameters that could be relevant at both the segmental and breath group level: duration, intensity, mean fundamental frequency, standard deviation of fundamental frequency, F1, F2, center of gravity, and spectral standard deviation. We took a subset of the segments, sampling equally from individual and segment manner, so that the number of segments would equal the number of breath groups, 105. We scaled the acoustic parameters and clustered the units using the function *eclust(hc_method* = *“ward.D2”, hc_metric* = *“spearman”)* in the *R* package *factoextra*^27^. Using the *factoextra* function, *fviz_silhouette()*, we ran silhouette analyses and calculated the average silhouette score for cluster sizes from two to eight. In a silhouette analysis, each unit is placed in a cluster and gets a silhouette value based on the distance to other units within its cluster and to other units in the next nearest cluster. Silhouette values range from − 1 to 1. Negative values mean the unit was likely misclassified, numbers closer to 1 suggest that the unit is in a tight and non-overlapping cluster, and a value close to 0 suggests the unit lies between two clusters^78,79^. For each cluster size, we obtained an average silhouette value. Units that are more basic would be expected to repeat and therefore cluster together more clearly which should lead to higher silhouette values. Units with subunits should show more acoustic overlap as they may have some subunits in common leading to lower silhouette values.

### Segmentation validation: clustering of budgerigar vocal units

For each budgerigar segment and syllable, we measured and extracted 21 acoustic variables (Table 1, Supplemental material).

For budgerigar song, we randomly selected a subset of segments (n = 840) and a subset of syllables (n = 840), sampling equally from each individual (60 was the minimum number of complex syllables collected from individual budgerigars). To help us assess if the success of the algorithm, we included simple syllables (n = 840) and random snippets extracted from complex syllables (n = 840). We included the random snippets to ensure better clustering of segments was not simply an artefact of shorter units. The random snippets were not truly random but were dependent on the “real” durations of segments. Using the start and end times of the segments stored in Praat TextGrids, we extracted segment durations from each complex syllable. We then randomized the order of the durations (Praat *Table* object function *Randomize rows*) and reinserted new segment boundaries based on the rearranged durations. While there is the possibility of the new boundaries aligning with actual divisions, particularly with short segments, the random boundaries are not dependent on acoustic transitions. As such, if they perform as well or better than our algorithm’s segmentation, then we can assume the algorithm failed in detecting segments.

For each unit, we used the measurements listed in Table 1 in the Supplemental material as input for the clustering.

As with the human data, we scaled the data for each acoustic parameter and performed a hierarchical clustering using the function *eclust(hc_method* = *“ward.D2”, hc_metric* = *“spearman”)* in the *R* package *factoextra*^27^. Using the *factoextra* function, *fviz_silhouette()*, we ran silhouette analyses for a range of cluster sizes. The minimum cluster size was 2 clusters and the maximum was 15.

We compared the mean silhouette widths for complex syllables, segments, and random snippits. Because a *k* of 2 had the highest silhouette widths for the segments, we ran summary statistics on the two clusters for a few acoustic variables (periodicity, intensity, duration, and Wiener entropy).

### Segmentation validations: using vocal units to predict population

We implemented a supervised random forest classification algorithm^80,81^ to assess the possibility that the acoustic cues in segments and syllables could be used by budgerigars to determine group or individual identity. We used the function *randomforest()* from the *randomForest* package and used the acoustic measurements listed in Table 1 in the Supplemental material. We trained four random forest models; the models varied in the type of acoustic input data (syllables vs. segments) and in the classification output (group vs. individual identity). Because some groups and individuals had more samples than others, we took a random subset of the data for each model. For the model classifying individuals using segment data, we used 500 segments from each individual. For the model classifying groups using segment data, we used 500 segments from each group. Because we had a much smaller number of syllables in the dataset, we took a sample size based on the group or individual with the smallest number of samples. For the model classifying individuals using syllable data, we took a random sample of 60 syllables from each individual. For the final model, group classification from syllable data, we took a random sample of 384 syllables from each group. We set the number of trees to create in the algorithm at 500 and we used three predictors at each node split.

To assess whether the algorithms performed above chance at classification, we used an exact binomial test, *binom.test()* in *R*, for each of the models. We corrected for multiple testing by using *p.adjust(method* = *“Holm”)* in *R.*

### Segmentation validation: syllable edge effects

To evaluate the effect of segment position on the acoustic signal, we divided the segments into three categories based on their relative position in the syllable: initial, medial, and final. We defined initial segments as the first segment of a syllable, final as the last, and the medial group included everything in between. We evaluated four acoustic measurements: mean fundamental frequency, duration, and intensity, and periodicity.

We used mixed effect models to assess whether segment position has an effect on the acoustic output. Mixed effect models, particularly generalized linear mixed models, allow for more flexibility and accuracy when analyzing non-normal data like ours which vary in samples per position, samples per individual, and number of individuals per group^82^. We used the *lme4*^83^ package in *R* with segment position and population as fixed effects. We included population as a covariate to better assess if the segment patterns occur independent of group. We included individual identity as a random effect. We used the *anova()* function in *R*’s base *stats* package to compare the model with a null model that excludes the fixed effect of segment position. We also compared the full model with a model in which the covariate group was removed. We used the *vif()* function in *car*^84^ to check for collinearity.

Visual assessment of the residuals for periodicity, mean fundamental frequency, and duration were all non-normal, so we used *lme4*′s *glmer()* function to fit a generalized linear mixed model (F0: Gaussian distribution with “log” link; Duration: an inverse Gaussian distribution with “identity” link; Periodicity: binomial distribution with “logit” link.) For intensity, the residuals were normal and homoscedastic, so we fitted a linear mixed model.

### Ethical compliance

All procedures performed in animals were conducted in accordance with local animal protection and housing laws and were approved by the ethical board of the behavioral research group in the faculty of Life Sciences at the University of Vienna (Approval Numbers 2015-005; 2018-019) or the Animal Care and Use Committee of the University of Maryland, College Park. Data collected from human subjects were taken from publicly available databases or were approved by the University of Vienna Ethics Committee (Approval Number 00063) and were conducted in line with the Declaration of Helsinki (1964).

## Supplementary Information

Supplementary Information.

## Data Availability

All data and code are available upon request via the corresponding author.
