# Supplementary material for: Universal principles underlying segmental structures in parrot song and human speech
Source: Sci Rep. 2021 Jan 12;11:776. doi: 10.1038/s41598-020-80340-y (PMC7804275; doi:10.1038/s41598-020-80340-y)
Supplement: Supplementary file 1 — Supplementary Information. [file 41598_2020_80340_MOESM1_ESM.pdf]

Universal principles underlying segmental structures in parrot song and human speech

Dan C. Mann[1,2]*, W. Tecumseh Fitch[2] , Hsiao-Wei Tu[3], & Marisa Hoeschele[2,4]

[1] Linguistics Program, The Graduate Center of the City University of New York, New York City, USA

[2] Department of Cognitive Biology, University of Vienna, Vienna, Austria

[3] Department of Psychology, University of Maryland, College Park, USA

[4] Acoustics Research Institute, Austrian Academy of Sciences, Vienna, Austria

* Corresponding author

daniel.mann@univie.ac.at

Supplemental material

Table 1| Acoustic parameters measured

Mean Fundamental frequency (F0): Frequency. Measured in Hertz (Hz). Result of (quasi) periodic vibration of labia (lateral tympaniform membranes; Larsen & Goller, 2002) in the syrinx (vocal source in avians). Measured by creating a pitch contour from Praat's *To Pitch (ac)…* function (along with all other F0 calculations) then calculating the mean frequency for the sound by using *Get mean…*. The advanced settings were the same as in the division algorithm, except that the minimum frequency (which determines the analysis window) was set to a standard species minimum of 500 Hz: *To Pitch (ac): 0, 500, 15, "no", 0.03, 0.45, 0.05, 0.15, 0.04, 10000*.
Minimum F0: Frequency. Hz. Lowest F0 value found in frequency contour. Praat *Pitch* object function: *Get minimum…*.
Maximum F0: Frequency. Hz. Highest F0 value found in frequency contour. Praat *Pitch* object function: *Get maximum…*.
F0 range: Frequency. Hz. Difference between maximum F0 and minimum F0.
Start F0: Frequency. Hz. F0 at unit beginning. Measured by taking the Praat *Pitch* object, starting from the onset of the sound and searching until Praat's F0 calculation was able to find evidence of voicing.
Mid F0: Frequency. Hz. F0 at the halfway point of the unit. Measured by finding the midpoint of the unit and then taking the F0 measurement from the Praat *Pitch* object.
End F0: Frequency. Hz. F0 at unit end. Measured by taking the Praat *Pitch* object, starting from the offset of the sound and searching backwards until Praat's F0 calculation was able to find evidence of voicing.
F0 slope: Frequency. Hz. The difference between the start and end F0. Negative numbers mean F0 falls over the course of the vocalization.
F0 slope/time: Frequency. Hz/msec. Slope corrected for duration. It is calculated as the difference between the fundamental frequency at the unit start and unit end divided by total duration.
Time-frequency excursion: Frequency/temporal. Hz. The sum of frequency modulations divided by the unit duration. Calculated by taking the Praat *Pitch* object and summing the absolute difference between each frequency measurement. The sum is then divided by the unit duration.
Jitter: Frequency. Percent. Perturbations/deviations in the fundamental frequency. Calculated with Praat's *PointProcess (periodic, cc)…* and *Get jitter (local)…* functions. I set *Period floor* at 0.0001, the *Period ceiling* at 0.00167, and *Maximum period factor* at 1.3.
Shimmer: Amplitude. Perturbations/deviations in amplitude. Praat *PointProcess* object function *Get shimmer (local)…*. Same input parameters as jitter, plus *Maximum amplitude factor* at 1.6.
Duration: Temporal. Seconds. Amount of time the unit lasts. Praat's *Get total duration...* gives a measurement in seconds so I converted to milliseconds by multiplying by 1000.
Time to maximum amplitude: Temporal. Percentage. Calculated by taking time point of the maximum amplitude (*Get time of maximum…*) in the unit and dividing by the total unit duration.
Periodicity: Quality. Present/Absent. If a (quasi)-periodic signal is present in the unit. Uses the output of Praat's *Pitch* object.

Wiener entropy: Spectral. Unitless. A measure of how much energy is spread across the sound spectra. I calculated Wiener entropy by dividing the sound into 10 msec windows, with a 9 msec overlap, and calculating the amount of energy at each frequency bin (100 Hz). I then calculated the geometric and arithmetic mean energy across the bins for each 10 msec window slice. I took the logarithmic score of the geometric mean divided by the arithmetic mean so that white noise (energy at all frequencies) is 0 and a pure tone is negative infinity. The final measurement is the mean Wiener entropy score for each window in the sound. Since Praat does not have a built in Wiener entropy function, we built our own (based on Gabriel J. L. Beckers Wiener entropy script: http://www.gbeckers.nl/pages/praat_scripts/wiener_entropy.praat_script).
Center of gravity: Spectral. Hz. The average frequency over the whole spectrum of a sound weighted by the spectrum. Center of gravity is calculated such that for a sine wave the center of gravity is the same as the frequency of the sine wave, while the center of gravity for white noise is half of the Nyquist frequency. I used Praat's *Spectrum* object function *Get centre of gravity…* with a *Power* setting of 2.
Standard deviation: Spectral. Hz. The standard deviation in the center of gravity. *Spectrum* object function *Get standard deviation…* with a *Power* setting of 2.
Skewness: Spectral. A measure of the symmetry in the spectral distribution, that is how different is the energy distribution above and below the center of gravity. *Spectrum* object function *Get skewness…* with a *Power* setting of 2.
Kurtosis: Spectral. A measure for how different the energy distribution across frequency bins (centered on the center of gravity) is from a Gaussian distribution. *Spectrum* object function *Get kurtosis…* with a *Power* setting of 2.
Intensity: Amplitude. dB. The acoustic correlate of loudness. Measured with *To Intensity…*

Table 2| Random Forest classification model success rates for budgerigar units

Unit/ Population	Success Rate	Chance Level	Binomial Test
Syllable – Group	71%	25%	p<0.001
Segment – Group	48%	25%	p<0.001
Syllable – Individual	40%	7%	p<0.001
Segment – Individual	24%	7%	p<0.001

Table 2. Binomial tests were performed by using *binom.test()* in R. We used *p.adjust(method = "Holm")* to correct for multiple comparisons.

Table 3| Group means (of individual means) by segment position

Group	Segment position	Intensity dB	Duration MSec	Periodicity %	F0 Hz
**A**	**Initial**	46.3 (±2.72)	4.99 (±0.75)	28.22 (±4.25)	2633 (±181)
N = 7	**Medial**	56.75 (±3.19)	6.13 (±0.58)	71.19 (±3.58)	2611 (±152)
	**Final**	52.51 (±3.37)	10.77 (±2.7)	49.79 (±6.89)	2081 (±110)
**B**	**Initial**	54.62	6.01	34.83	2442
N = 1	**Medial**	62.35	7.86	66.66	2479
	**Final**	58.05	10.53	44.77	1828
**C**	**Initial**	48.72 (±1.89)	7.16 (±1.03)	39.57 (±5.74)	1705 (±208)
N = 2	**Medial**	59.27 (±1.59)	6.43 (±0.73)	79.24 (±1.57)	2231 (±105)
	**Final**	55.47 (±3.24)	6.82 (±0.97)	50.44 (±0.36)	2118 (±206)
**D**	**Initial**	44.57 (±3.7)	5.82 (±1.93)	42.07 (±15.76)	2578 (±50)
N = 4	**Medial**	52.94 (±3.95)	6.82 (±1.07)	78.11 (±1.14)	2524 (±132)
	**Final**	50.51 (±6.23)	16.97 (±5.63)	68.49 (±4.88)	2085 (±240)

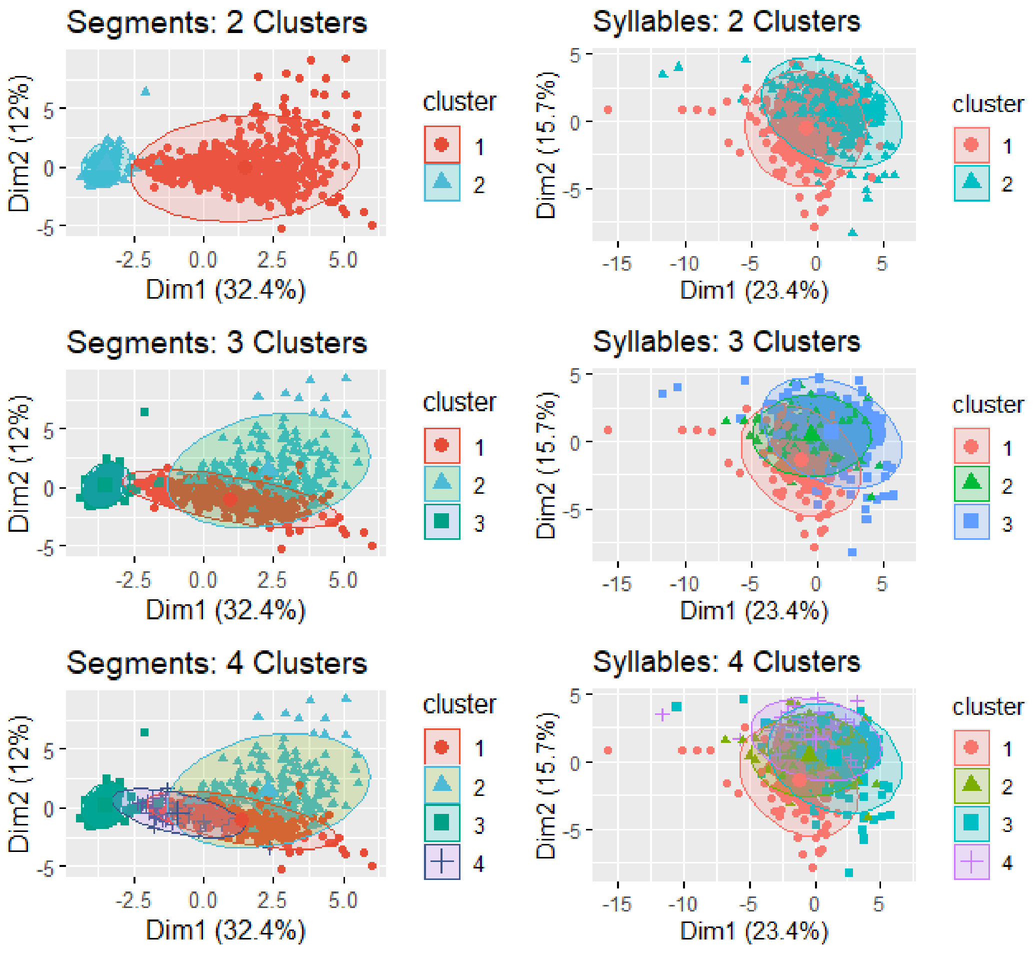

Fig 5. Budgerigar warble song segment and syllable cluster sizes of 2, 3, and 4. The function fviz_cluster() in the *factoextra*[69] package performs a principal components analysis

and plots the data points with the cluster information. Ellipses represent a multivariate normal distribution with a 0.95 confidence interval.

Alternative Random Forest Implementation. To assess whether segments or syllables were better at predicting individual and group membership, we built four random forest models: predicting individuals from syllable data, predicting individuals from segment data, predicting groups from syllable data, and predicting groups from segment data. We implemented a supervised random forest classification algorithm using the function *randomforest()* from the *randomForest* package. Data labels were either individual or group and we used the variables in Table 1 as input for the models. We set the number trees to grow at 500 and we used three predictors at each node split.

For each model, we split the full dataset equally for each individual so that we had four datasets of 840 units (each individual had 60 simple syllables, 60 complex syllables, and 60 segments). We split the data into 608 units for training and left 232 units for the testing set.

Table 4| Random Forest classification model success rates for budgerigar units

Unit/ Population	Success Rate on Testing Set	Chance Level	Binomial Test
Syllable – Group	78 %	25%	$p<0.001$
Segment – Group	62.5%	25%	$p<0.001$
Syllable – Individual	39.7%	7%	$p<0.001$
Segment – Individual	19.1%	7%	$p<0.001$

Table 4. Binomial tests were performed by using *binom.test()* in R. We used *p.adjust(method = "Holm")* to correct for multiple comparisons.

Clustering and segment classes.

We assessed how clusters of simple syllables, complex syllables, and segments compared to each other and whether broad unit classes could be found across all groups. Based on visual inspection of the budgerigar spectrograms, we expect segments and simple syllables to overlap in acoustic space.

We created three datasets, one for each unit type: simple syllables, complex syllables, and segments. We used a subset of the full dataset such that each individual had 60 simple syllables, 60 complex syllables, and 60 segments (840 samples for each of the three datasets). Each unit had measurements for the 21 acoustic variables listed in the supplementary Table 1. We then used the *eclust()* function in the *factoextra* package to perform a hierarchical clustering on each dataset using the standardized acoustic measurements (*hc_method* = *"ward.D2", hc_metric* = *"spearman", stand* = *TRUE*). We set the cluster size, *k*, to 4. We chose 4 both to have equal numbers of clusters for each of the three unit types and because the largest drop off in silhouette scores was after four clusters (for simple syllables).

Units

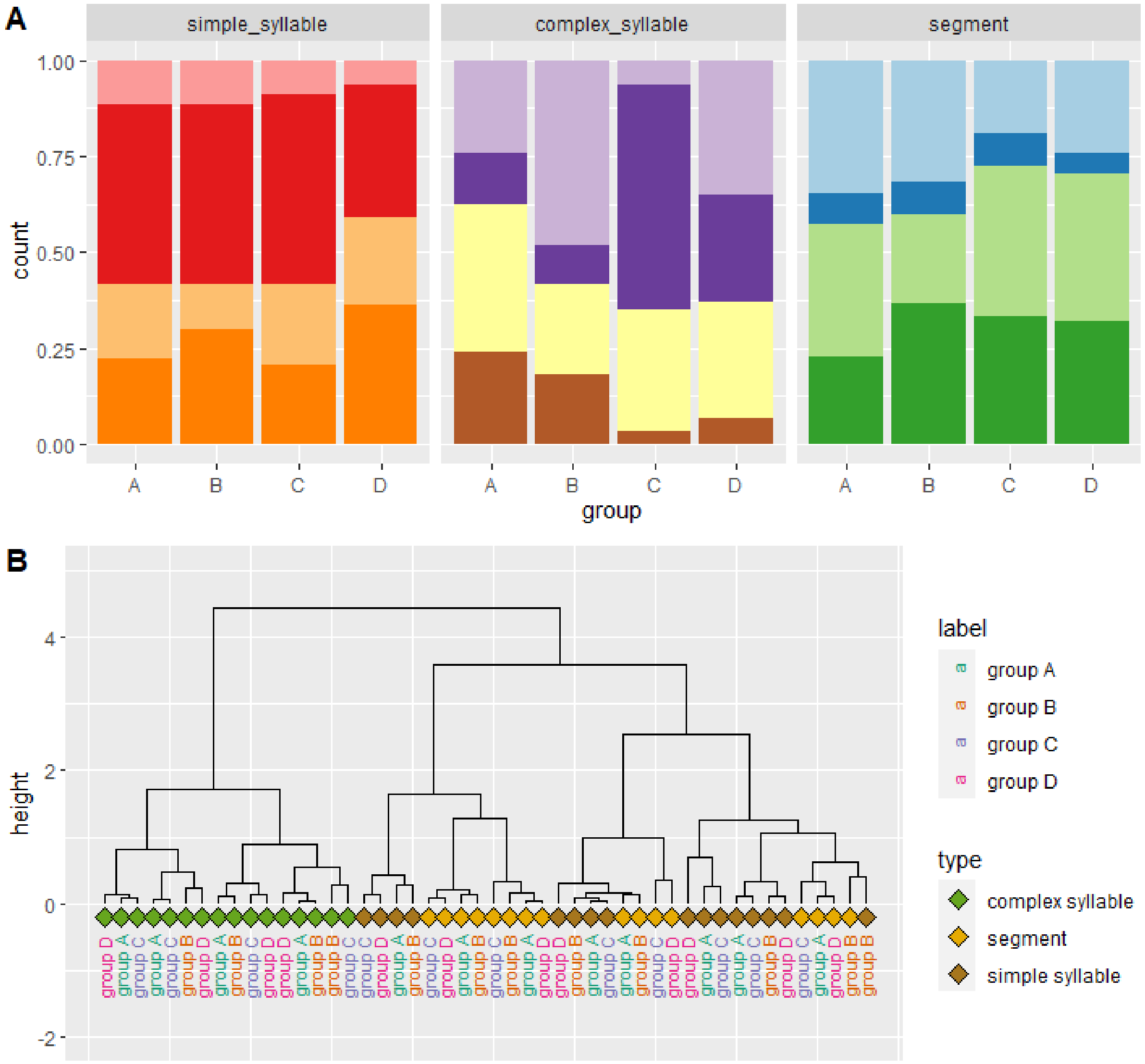

Fig 6. Units across populations. (a) The four clusters created for each unit type are distributed across all groups. (b) Hierarchical clustering on the mean scores of those clusters shows acoustic overlap between segments and syllables. The complex syllables form their own cluster separate even from the segments that comprise them. The units don't cluster by group.

All cluster types exist across all groups. We grouped the data points by unit type, cluster, and population (group) and calculated a mean score. Each group of budgerigars has twelve mean values: mean values for four clusters in the three unit types (total of 48 observations). We applied another hierarchical cluster (same details as above without specifying *k*) to the 48 mean scores.
